# The platelet-related genes associated with the prognosis of HCC by regulating cycling T cell and prolif-TAMs

**DOI:** 10.1016/j.heliyon.2024.e26798

**Published:** 2024-02-27

**Authors:** Chenjia Peng, Ying Wang, Hengbo Zhang, Ping Chen

**Affiliations:** a School of Mathematics and Computational Science, Hunan First Normal University, Changsha, 410205, PR China; b The National & Local Joint Engineering Laboratory of Animal Peptide Drug Development, College of Life Sciences, Hunan Normal University, Changsha, 410081, PR China; c Physical Education Department, First Hunan Normal University, Changsha, 410081, PR China

**Keywords:** Platelet, HCC, Prognosis, Risk model, Immune infiltration

## Abstract

Accumulating evidence highlighted the important roles of platelets in the prognosis and progression of various tumors. Nevertheless, the role of platelet-related genes (PRGs) in HCC remains limited. In this work, 92 differentially expressed PRGs were described in HCC using TCGA and ICGC databases. Then, based on the different expressions of PRGs, we explored two subtypes and developed the PRGs prognostic signature in HCC. The PRGs signature was an independent prognosis factor associated with immune cell infiltration in HCC. Furthermore, two external validation sets verified the expression and prognosis of the PRGs signature gene in HCC. Finally, scRNA-seq analysis demonstrated that the signature genes (CENPE and KIF2C) were mainly expressed in cycling T cells and prolif-TAMs. Enrichment analysis showed that CENPE and KIF2C regulated the cell cycle and p53 pathways in these cells. In conclusion, this study builds the PRGs-related risk signature of HCC and reveals the potential mechanism by which these signature genes regulate the immune microenvironment in HCC.

## 1 Introduction

Hepatocellular carcinoma (HCC) is a worldwide common malignancy with a high incidence and mortality rate. Despite the improved treatment for HCC, its five-year survival rate is only 5%–7%, and the recurrence rate is up to 60–70%, leading to the major causes of cancer-related death worldwide [1]. So, it is urgent to reveal novel biomarkers for predicting HCC prognosis and develop feasible therapeutic strategies for HCC.

Platelets, one of the anucleate blood cells in humans and animals, are created in the bone marrow and then circulate to the whole body to involve hemostasis and thrombosis [2]. Moreover, accumulated studies indicated the more comprehensive functions of platelets in physiological and pathological processes, including lymphatic vessel development, angiogenesis, atherosclerosis, and carcinogenesis [[3], [4], [5]]. Over 100 years ago, thrombus accompanying cancer was first time observed [6]. Subsequently, the number of platelets was associated with the poor prognosis of various cancers, especially the platelet-to-lymphocyte ratio (PLR) function as the prognosis biomarker in several cancers [[7], [8], [9]]. Emerging studies showed that platelets regulate carcinogenesis, metastasis and progression [10]. Studies also implied the role of platelets in regulating the immune microenvironment and predicting the immunotherapy response to tumor progression [11,12]. Dysregulation of platelet count and function was described in chronic liver disease [13,14]. In HCC, platelet count and PLR were reported to be related to the prognosis of patients [6,15]. The activated platelets promote the progression of HCC by regulating the tumor immune microenvironment [16]. Anti-platelet therapy was reported to improve HCC outcomes [[17], [18], [19]]. However, whether platelet-related genes (PRGs) could be the prognostic marker of HCC prognosis remains unclear.

In this study, PRGs were the genes associated with platelet biological functions and were collected from GSEA gene sets. We analyzed the expression and genetic alterations of PRGs in HCC samples from various datasets (including GEO, ICGC and TCGA) and explored PRGs subtypes and PRGs prognosis signature, which were associated with survival and the immune microenvironment. Finally, the scRNA dataset revealed the expression and regulation of signature genes on cycling T cells and prolif-TMA in HCC.

## 2 Results

### 2.1 Platelet-related genes in HCC

**Fig. 1:**
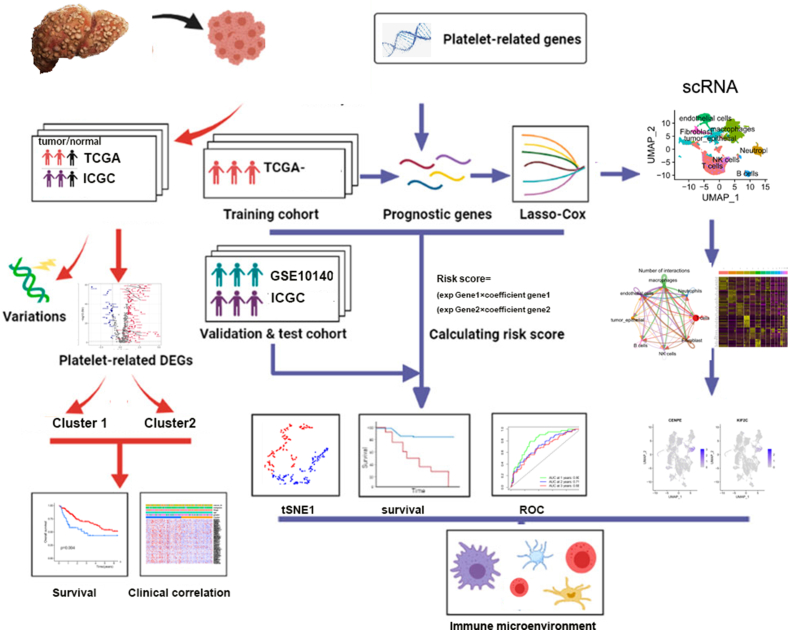
Flow chart.

**Fig. 2:**
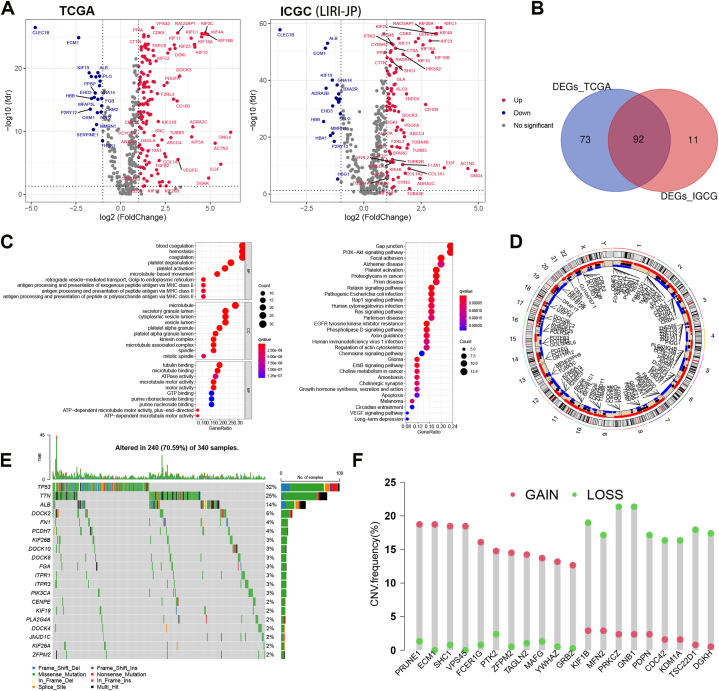
The landscape of PRGs in HCC. A, The volcano plot of PRGs in HCC from TCGA and ICGC database. B, The Veen graph of differentially expressed PRGs in HCC from TCGA and ICGC. C, The GO and KEGG enrichment analysis. D, The location of PRGs on the chromosome. E, The Waterfall plots displaying the mutation landscapes of PRGs in HCC from TCGA. F, The CNV values of PRGs in HCC from TCGA.

Here, 480 platelet-related genes (PRGs) that were correlated to biological functions of platelet were obtained from the GSEA database by the keyword “platelet” (Table S1). Then, we analyzed the expression of PRGs in HCC using TCGA and ICGC databases. The flow chart of this work is illustrated in Fig. 1. Fig. 2A shows volcano plot of PRGs between normal liver tissues and HCC. And 92 differentially expressed PRGs (*p*-value <0.05 and |log2FC| > 1) overlapped in HCC from TCGA and ICGC databases (Fig. 2B). The GO and KEGG enrichment analysis showed that these differentially expressed PRGs were enriched in platelet activation, Ras signaling pathway, Gap junction, VEGF signaling pathway, chemokine signaling pathway, and so on (Fig. 2C). Moreover, the CNV status and variations of PRGs were analyzed in Fig. 2D–F. Fig. 2D shows the location of PRGs on the chromosome. The waterfall plots showed that the PRGs, including TP53, TNN and ALB, possessed highly frequent mutations in HCC (Fig. 2E). Additionally, the CNV alteration showed that PRUNE1, ECM1, SHC1 and VPS45 had significant copy number amplification, while PRKCZ, GNB1 and KIF1B had significant copy number deletion (Fig. 2F).

### 2.2 PRGs-based classification in HCC

**Fig. 3:**
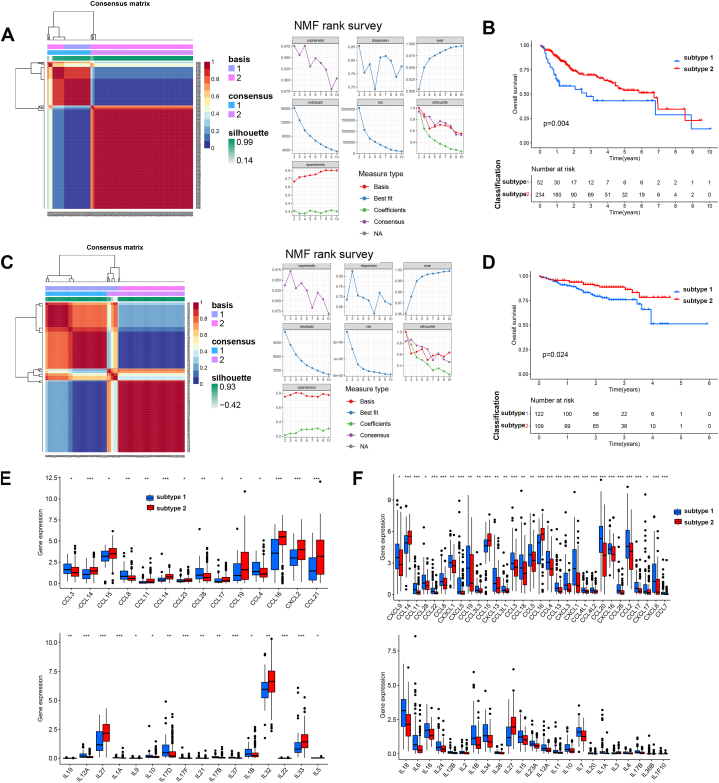
The PRGs-related subtypes in HCC. A, The NMF classification analysis of HCC from TCGA. B, The survival analysis of HCC patients from Subtype 1 and 2 using the TCGA dataset. C, The NMF classification analysis of HCC from ICGC. D, The survival analysis of HCC patients from Subtype 1 and 2 using the ICGC dataset. E, The heatmap of PRGs using TCGA dataset. F, The heatmap of PRGs using ICGC dataset. G, The cytokine and Chemokines expression in 2 Subtype from TCGA datasets. H, The cytokine and Chemokines expression in 2 Subtype from ICGC datasets. *, *p* < 0.05; **, *p* < 0.01; ***, *p* < 0.001.

Next, based on the 92 differentially expressed PRGs, the HCC patients from the TCGA dataset were classified into two distinct subtypes using NMF analysis (Fig. 3A). The patients in subtype 1 (*N* = 52) showed poor survival compared to those in subtype 2 (*N* = 234) (Fig. 3B). Similar results were confirmed using HCC samples from the ICGC database (Fig. 3C). The patients in subtype 1 (*N* = 121) showed poor survival compared to those in subtype 2 (*N* = 109) (Fig. 3D). The relationship between clinical characters (grade, stage, T, N, M age, and so on) and subtypes is shown in Fig. 3E and F. However, no significant correlation was observed. The heatmap showed that PRGs expression was much higher in subtype 1 than in subtype 2.

Studies showed that the activated platelets promote the progression of HCC by regulating the tumor immune microenvironment [16]. So, we analyzed the relationship between PRGs classification and immunity. Moreover, the immune analysis showed distinct immune function and immune cell infiltration in HCC (Figs. S1 and S2). Considering the key role of cytokines and chemokines in platelets-mediated immune regulation, the cytokines and chemokines expression were analyzed in two PRGs subtypes. Fig. 3G and H showed that cytokines and chemokines, including CCL3, CCL4, CCL15, CCL8, CXCL2 and IL27, were downregulated in subtype 1 than that in subtype 2. CCL19 and CCL14 were upregulated in subtype 1 than that in subtype 2 (Fig. 3G and H).

### 2.3 PRGs-based risk model of HCC

**Fig. 4:**
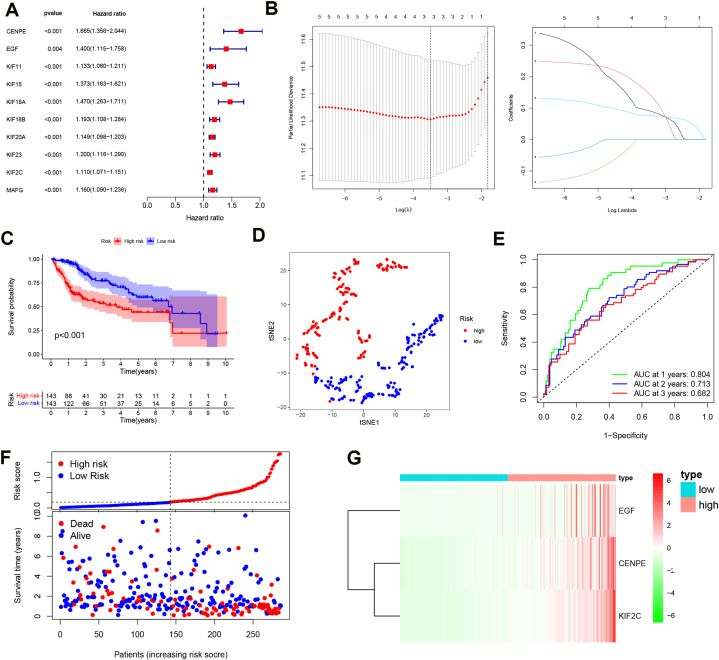
PRGs prognostic signature of HCC in the training TCGA dataset. A, The univariate Cox analysis. B, The LASSO Cox regression analysis. C, The survival analysis of HCC patients with high/low-risk scores. D The t-SNE analysis of HCC patients with high/low-risk scores. E, The ROC curve analysis. F, The distribution of the risk score. G, The heatmap of PRGs signature genes.

We constructed the PRGs prognostic signature based on the 92 PRGs using TCGA datasets. As shown in Fig. 4A, univariate Cox analysis identified that ten genes were associated with the overall survival of HCC patients in TCGA datasets (with *p* < 0.01, HR > 1.1). Next, a PRGs prognostic signature was constructed using LASSO Cox regression analysis (Fig. 4B). The risk score = CENPE*0.0916+ EGF*0.1464+ KIF2C*0.0761. The expression and prognosis role of PRGs signature genes were investigated using the Wilcoxon test and Kaplan–Meier (K–M) analysis (Fig. S3). We found that HCC patients in high-risk groups had a poor prognosis (Fig. 4C). The t-SNE analysis showed a distinct high/low-risk group (Fig. 4D). Moreover, the ROC curve indicated that the established prognostic model had excellent predictive efficacy with the AUC 0.804 for 1 year, 0.713 for 2-year, and 0.682 for 3-year survival (Fig. 4E). High-risk HCC patients a higher mortality (Fig. 4F). The HCC patients with higher risk scores showed higher expression levels of EGF, CENPE and KIF2C (Fig. 4G).

**Fig. 5:**
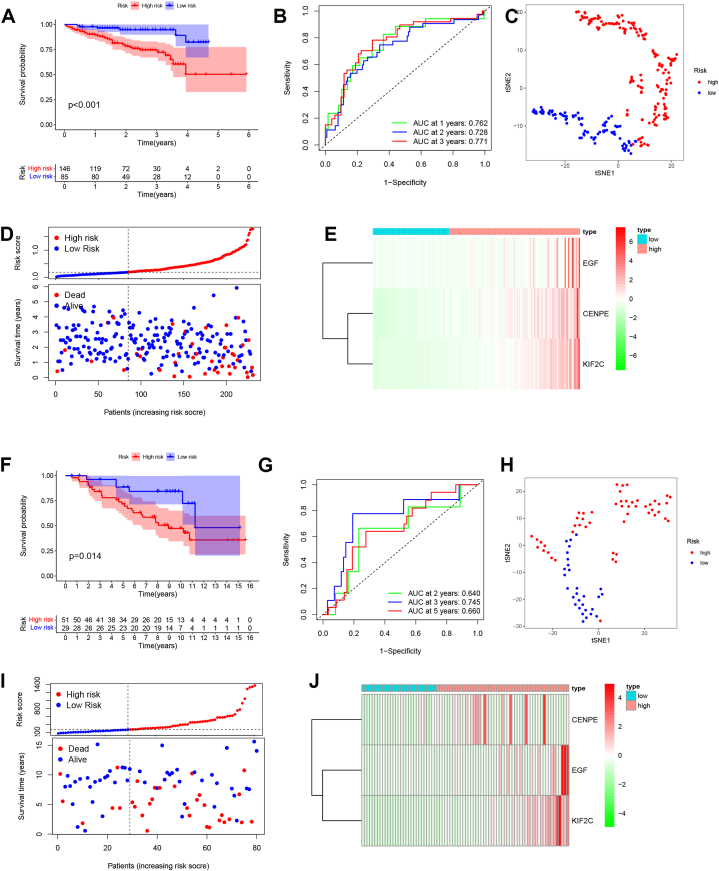
PRGs prognostic signature was verified in the ICGC and GEO datasets. A, The survival analysis of HCC patients with high/low-risk scores in the ICGC dataset. B, The ROC curve analysis using the ICGC dataset. C, The t-SNE analysis using ICGC dataset. D, The distribution of the risk score using the ICGC dataset. E, The heatmap of PRGs signature genes in ICGC dataset. F, The survival analysis of HCC patients with high/low-risk scores in the GSE10140 dataset. G, The ROC curve analysis using the GSE10140 dataset. H, The t-SNE analysis using the GSE10140 dataset. I, The distribution of the risk score using the GSE10140 dataset. J, The heatmap of PRGs signature genes in the GSE10140 dataset.

Next, we verified the risk signature using the ICGC and GEO datasets. As shown in Fig. 5A, the high risk showed a poor prognosis in the ICGC dataset and GSE10140 dataset (Fig. 5A and F). The AUC was 0.762 for 1 year, 0.728 for 2-years, and 0.771 for 3-years of survival in the ICGC dataset (Fig. 5B). The AUC was 0.64 for 2 years, 0.745 for 3-years, 0.660 for 5-years survival in the GSE10140 dataset (Fig. 5G). The distinct high/low-risk groups were observed in the ICGC and GEO datasets using t-SNE analysis (Fig. 5C and H). The landscape of risk score and PRGs signature genes were also confirmed in the ICGC dataset and GSE10140 dataset. The results showed that more HCC patients with a high-risk score were dead, and HCC patients with higher-risk scores showed higher expression levels of EGF, CENPE and KIF2C (Fig. 5D, E, I and J).

We next analyzed the relationship between the PRGs subtype and PRGs risk signature using ICGC and TCGA datasets. As shown in Fig. S4A, more patients in subtypes 1 were grouped in the high-risk group and were dead, and most patients were grouped in the low-risk group and survived. Moreover, the heatmap revealed that the PRGs signature was associated with the stage in HCC (Fig. S4B). The relationship between PRGs-based subtypes/risk model and TBM, RNAss using the TCGA dataset. The subtype 1 and high-risk group patients showed more mutations (Fig. S4C), and higher TBM and RNAss, and the risk score correlated with TBM and RNAss (Fig. S4D).

### 2.4 PRGs signature is an independent prognostic signature

**Fig. 6:**
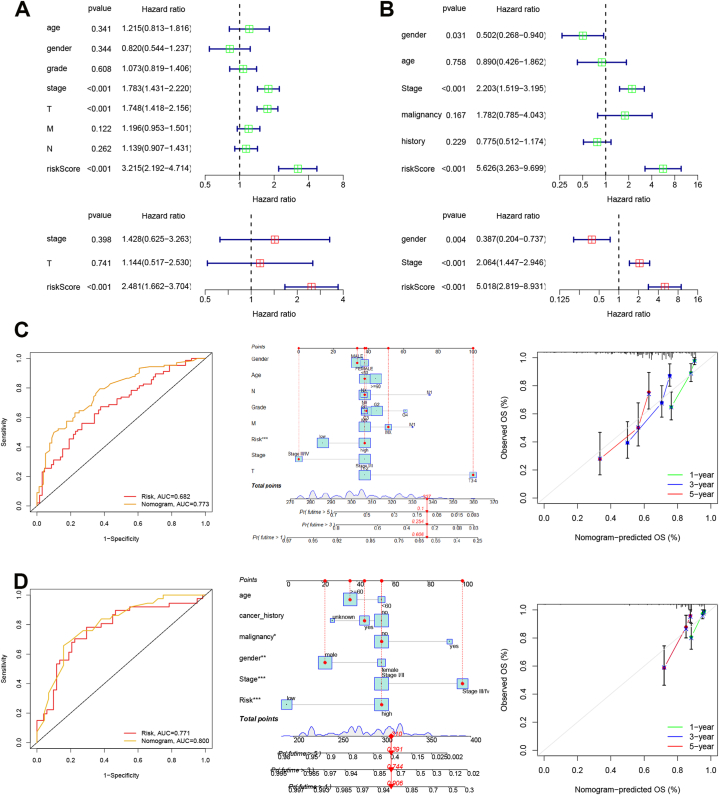
PRGs signature is an independent prognostic signature. A, Univariate and multivariate analysis for TCGA dataset. B, Univariate and multivariate analysis for ICGC dataset. C, The nomogram model, ROC cure, calibration plots for predicted probability in TCGA dataset. D, The nomogram model, ROC cure, calibration plots for predicted probability in ICGC dataset.

Next, the univariate/multivariable Cox analyses revealed that risk score was the independent prognostic factor in HCC (Fig. 6A and B) using TCGA and ICGC datasets. The risk score and clinical characters were collected for the nomogram model to estimate the probability of 1-, 3-, and 5-year OS (Fig. 6C and D). The ROC values for the nomogram were 0.733 and 0.8 in the TCGA and ICGC datasets, respectively. The consistency between predicted and actual survival rates was assessed using calibration curves and revealed the favourable accuracy of the nomogram model in 1,3,5-year survival rates (Fig. 6C and D).

To further verify the expression and prognosis of PRGs signature genes in HCC, we detected the expression of CENPE, EGF and KIF2C using GSE76427 GSE14520 datasets. As shown in Fig. S5, CENPE, EGF and KIF2C expression were robustly upregulated in HCC tissues compared to normal liver tissues. Moreover, the HCC patients were divided into high/low expression groups based on the median value of gene expression. As shown in Fig. S6, patients with high expressed CENPE, EGF and KIF2C showed poor prognosis in HCC.

### 2.5 PRGs signature correlates with immune in HCC

**Fig. 7:**
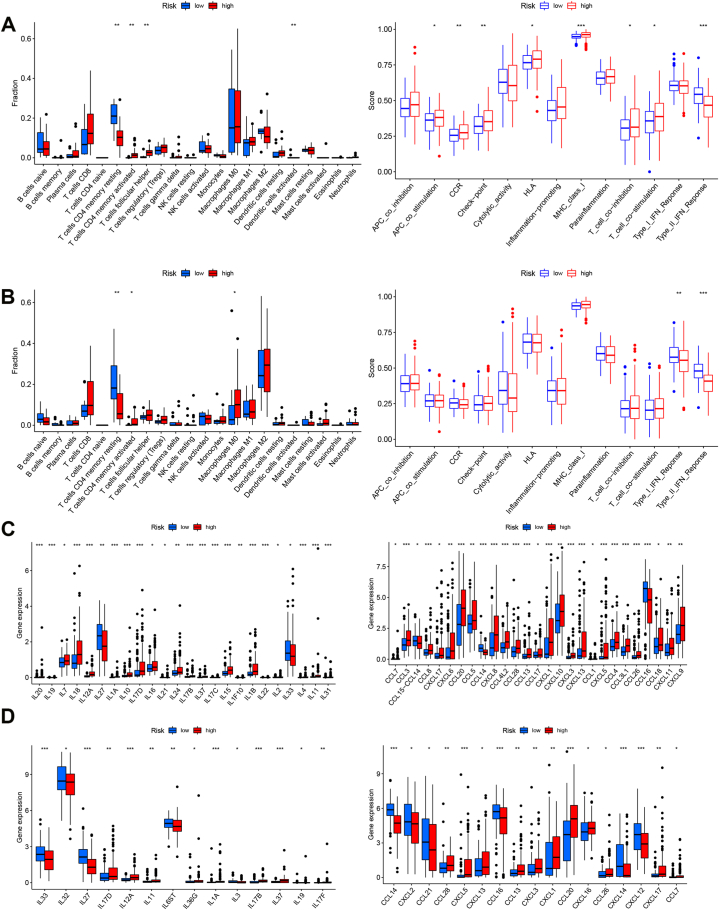
PRGs signature and the immune microenvironment in HCC. A, The immune infiltration and immune function in the high/low group in the TCGA dataset. B, The immune infiltration and immune function in the high/low group in the ICGC dataset. C, The cytokine and chemokines expression in HCC patients with high/low-risk signature from TCGA datasets. D, The cytokine and chemokines expression in HCC patients with high/low-risk signature from ICGC datasets. *, *p* < 0.05; **, *p* < 0.01; ***, *p* < 0.001.

We following analyzed the correlation between immune and PRGs signature. ssGSVA was used for the estimation of immune infiltration and immune function. As shown in Fig. 7A and B, the CD4 memory-resting T cells decreased and the CD4 memory-activated T cells increased in the high-risk group. The immune function analysis showed that high-risk HCC patients had lower Type II IFN response. Next, the immune infiltration was further assessed using QUANTISEQ, CIBERSORT, and XCELL. Less immune cell infiltration and higher cytokines and chemokines expression were observed high-risk group (Fig. S6, Fig. 7C and D).

Moreover, the checkpoint (LAG3, CTLA4, PDCD1, and CD70) and (CD44, CD27, PDCD1, and ICOS) were evidently upregulated in the high-risk group and subtype 2 group in the TCGA dataset and IGCG dataset, respectively (Figs. S7A and B).

### 2.6 ScRNA analysis revealed the expression and regulation of signature genes on the immune

**Fig. 8:**
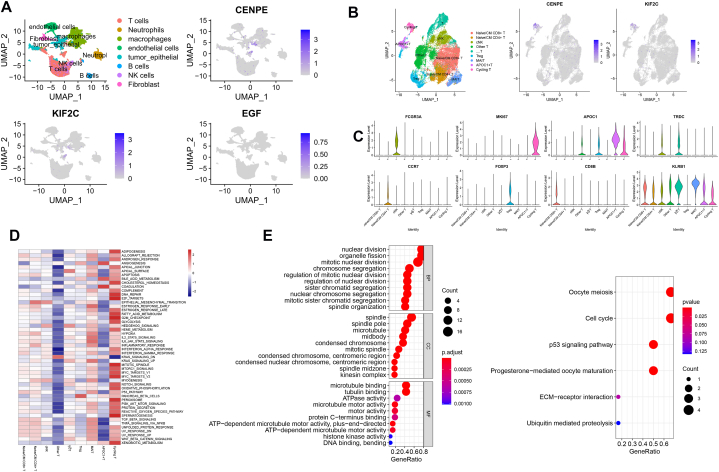
The scRNA transcriptomic analysis of HCC. A, UMAP plots reveal the cells with signature gene expression in HCC tissues. B, UMAP plots reveal the T cell subtypes with signature gene expression. C, The expression of marker genes in T cell subtypes. D, The GSVA analysis reveals the pathways in T cell subtypes. E, The GO and KEGG enrichment analysis of CENPE and KIF2C-related genes in cycling T cells.

Next, we identified the cells with signature genes’ expression using scRNA datasets. As shown in Fig. 8A and Fig. S8A, eight cell clusters were identified using umap analysis. CENPE and KIF2C were highly expressed in T cells and macrophages. No EGF expression was detected in the scRNA dataset. The cellchat analysis revealed the communication between T cells/macrophages and tumor/epithelial cells (Fig. S8B). We next analyzed the subcluster of T cells using UMAP analysis (Fig. S8C and D). Among T cells, nine subclusters (including Naive/CM CD4^+^ T, T reg, Naive/CM CD8^+^ T, mucosal-associated invariant T cells (MAIT), cNK, Cycling T, APOC1+T, γδT, and other T cells) could be identified basing on the known marker genes as previous described [20]. The gene expression in the nine populations is shown in Fig. 8C. We found that the CENPE and KIF2C were mainly expressed in Cycling T cells Fig. 8B. The GSVA analysis showed that the DNA repair pathway, G2M checkpoint pathway, glycolysis pathway, p53 pathway and inflammation-related pathways were significantly upregulated in cycling T cells (Fig. 8D). Next, we detected the CENPE and KIF2C-related genes in cycling T cells using Pearson analysis. The enrichment analysis showed that CENPE and KIF2C-related genes were involved in the cell cycle and p53 pathways (Fig. 8E).

**Fig. 9:**
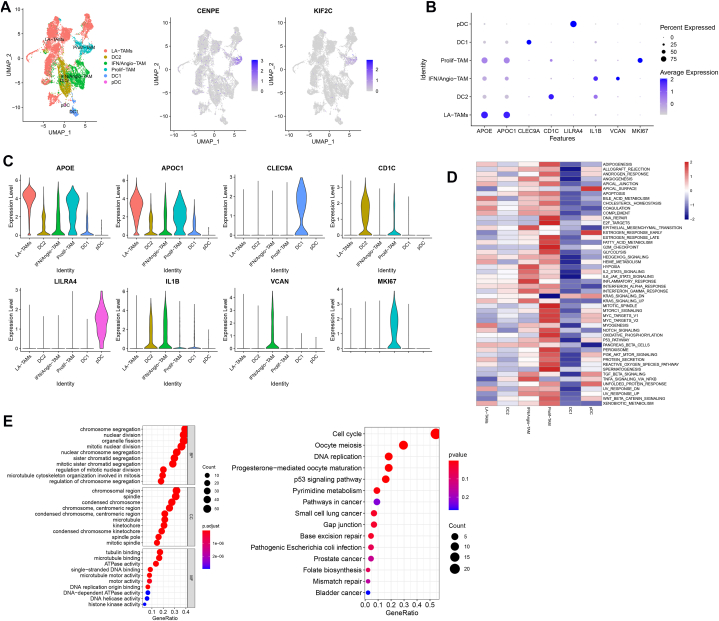
The scRNA transcriptomic analysis reveals signature gene expression in TAMs of HCC. A, UMAP plots reveal the macrograph subtypes with signature gene expression. B, The expression of marker genes in macrograph subtypes. C, The GSVA analysis reveals the pathways in macrograph subtypes. D, The GO and KEGG enrichment analysis of CENPE and KIF2C-related genes in Prolif-TAM.

We next identified the subtype of macrophages that expressed CENPE and KIF2C. As shown in Fig. 9A and Fig. S9A, six subtypes of macrophages (including lipid-associated TAMs (LA-TAMs), DC1, DC2, pDC, interferon-primed/proangiogenic TAMs (IFN/Angio-TAMs), inflammatory cytokine-enriched TAMs (Inflam-TAMs), proliferating TAMs (Prolif-TAMs)) were detected based on the marker gene as previous described [21]. The expression of the marker genes in the subtypes is shown in Fig. 9C. We found that the CENPE and KIF2C were mainly expressed in prolif-TAM cells (Fig. 9B). The GSVA analysis showed that the DNA repair pathway, E2F-target, glycolysis pathway, p53 pathway, peroxisome, oxidative phosphorylation pathways and inflammation-related pathways were significantly upregulated in prolif-TAM (Fig. 8D). Next, we detected the CENPE and KIF2C-related genes in prolif-TAM using Pearson analysis. The enrichment analysis showed that CENPE and KIF2C-related genes were involved in the cell cycle, DNA replication and p53 pathways (Fig. 8E). In conclusion, these results indicated that CENPE and KIF2C are involved in the progression of HCC by regulating the function of cycling T cells prolif-TAMs.

## 3 Discussion

HCC is a common lethal malignancy with a high mortality rate worldwide. Increasing evidence has revealed platelets' prognostic role in tumors, including HCC. Moreover, some platelet-related genes were reported to contribute to the prognosis and progression of tumors. However, the landscape of platelet-related genes and their prognostic value for HCC remains unclear. In this study, we described PRGs-based subtypes and risk signatures and explored the potential correlation between signature genes and immune microenvironments in HCC.

Platelets, small anucleate cells, are critical for primary hemostasis [22]. Accumulating studies identified the critical roles in various tumor progression. The number of platelets and PLR was reported as the prognosis biomarker in several cancers, including HCC [15]. Platelets were also implicated in multiple phases of a tumor by supporting survival and promoting growth and metastasis [23,24]. Platelet RNA profile was also described as a biomarker of tumor and promoted tumor progression [23]. α-Granules and hepatocyte growth factor (HGF) released from platelets could influence tumorigenesis [25]. In this work, 92 PRGs, enriched in platelet activation, Ras signaling pathway, and chemokine signaling pathway, were differentially expressed in HCC from TCGA and ICGC datasets with different CNV status and variations.

In recent years, accumulating studies demonstrated the prognostic role of platelet-related genes (PRGs) and PRGs-mediated molecular subtypes mediated, which were associated with the tumor microenvironment characteristics [[26], [27], [28]]. In our study, based on different expression PRGs, we explored two PRGs-based subtypes and developed and verified the 3 PRGs (CENPE, EGF and KIF2C) risk signature in HCC. The patients from subtype 1 and a high-risk group showed poor prognosis, higher mutation, and tumor mutation burden. It is worth noting that Centrosome-associated protein E (CENPE), a kinetochore protein, was involved in the progression of various tumors [29,30]. Epidermal growth factor (EGF) could be released by platelets, contributing to wound healing [31,32] and tumor growth [33]. KIF2C, a mitotic centromere-associated kinesin, was reported as a prognostic biomarker and related to immune Infiltration in various tumors [34,35]. Increased KIF2C was observed in HCC, and it was associated with the pathogenesis of HCC via mTORC1 signaling [36]. In this study, these three PRGs were upregulated, and as the risk genes for HCC, the PRGs signature based on these genes was also predicted as the independent factor of HCC.

Studies showed that the immune microenvironment and immune escape are the most critical factors for the unsatisfied clinical outcome of patients with HCC [37]. scRNA-Seq of HCC also revealed the development in subsets of immune cells, including tumor-associated macrophages, T cells, dendritic cells, innate lymphoid cells, and so on [38,39]. The immune cells, including macrophages, tissue-resident memory T-cells, and CD8^+^ T cells, were associated with the prognosis of HCC [40]. Immunotherapy is emerging as an effective strategy for tumor treatment, including HCC [41]. Increasing evidence demonstrated platelets as drivers of immune responses. Platelets function as a regulator of macrophages, NK cells and T cells [42]. In the progression of various tumors, platelets secrete inflammatory factors (such as TNFα and IL-6) that contribute to the regulation of the immune microenvironment [24]. Additionally, anti-platelet functions as a therapeutic strategy for HCC development [6]. Here, the PRGs subtype and PRGs signature correlates with the immune dysregulation in HCC. Moreover, scRNA revealed the expression of CENPE and KIF2C in cycling T cells and prolif-TAM. The cycling liver-resident T was first observed by Zhao et al., which expressed replicating markers such as MKI67 [20]. The prolif-TAM was a subtype of tumor-associated macrophages (TAM) characterized by highly expressed MKI67. CENPE and KIF2C-related genes in cycling T cell and prolif-TAM were enriched in cell cycle, DNA replication, and p53 pathways, implying the regulation of these genes on the proliferation of cycling T cell and prolif-TAM. Although the function of cycling T and prolif-TAM are unclear, the GSEA results revealed potential immunoregulation of these cells in HCC. We speculated that CENPE and KIF2C related to tumorigenesis of HCC by regulating cycling T cells prolif-TAMs. However, this hypothesis should be verified in further experiments. Given the signature genes are only expressed in cycling T and prolif-TAM cells, we speculated that the signature presents the infiltration of these two cells which is associated with HCC prognosis.

However, there were also several limitations in this study. First, based on the retrospective data from the public database, we constructed and validated the PRGs signature, but a larger prospective study is needed to confirm its prognostic robustness and clinical utility. Secondly, the expression of PRGs signature genes in cycling T and prolif-TAM need to be further verified by immunohistochemistry. Finally, the specific mechanism by which PRGs signature genes regulated the function of cycling T and prolif-TAM is further needed.

### 3.1 Conclusions

In conclusion, we systematically evaluated the landscape of PRGs in HCC, constructed PRGs-based subtype and prognosis signature with immune infiltration characteristics, and revealed the potential mechanism by which these signature genes regulated the immune in HCC.

## 4 Materials and methods

### 4.1 Data

TCGA, ICGC, and GEO (GSE10140) databases were consulted for gene expression data (FPKM) and clinical characteristics of HCC and normal tissues (Tables S2 and S3). 374 HCC and 50 normal tissues from TCGA (https://portal.gdc.cancer.gov/), 240 HCC and 197 normal tissues from ICGC-LIRI-JP (Liver Cancer - RIKEN, JP, https://dcc.icgc.org/), 80 HCC and 82 normal tissues from GSE10140 (https://www.ncbi.nlm.nih.gov/geo/query/acc.cgi?acc=GSE10140). The mutation data of HCC patients were downloaded from the TCGA database. Moreover, the expression and prognosis of PRGs signature genes were verified in the GSE76427, GSE10143, and GSE14520 datasets (https://www.ncbi.nlm.nih.gov/geo/). Finally, the scRNA of HCC (GSE202642) was downloaded for the verification of signature genes.

Four hundred eighty platelet-related genes (PRGs) were collected from GSEA (gene set enrichment analysis, http://www.gsea-msigdb.org/gsea/downloads.jsp). The differential expression analysis was performed using R “edgeR” packages [26]. The volcano plot of differentially expressed PRGs was performed using the R “ggplot2” package [43].

GO and KEGG were performed for function enrichment using the R package “clusterProfiler” [44].

The varied characteristics of PRGs were performed using the “RCircos” package, and the mutation analysis was performed using “maftools” as previously described [45].

### 4.2 NMF classification model

Based on the overlapped differentially expressed PRGs, non-negative matrix factorization (NMF) was performed for clustering analysis. 286 HCC patients and 231 HCC patients from TCGA and ICGC datasets with complete clinical information were used for further analysis. The PRGs expression and overall survival (OS) of the TCGA or ICGC dataset were applied to two distinct PRGs-based classifications using the “NMF” R package as previously described [46,47].

### 4.3 Platelet-related prognosis signature

With the TCGA dataset (train), we used the "glmnet" package to shrink candidates to construct the most suitable signature using univariate Cox regression. A Kaplan–Meier analysis and ROC analysis were performed to evaluate the predictive ability of PRGs signatures. These packages were “survival” and “timeROC”.

Based on the median value of the risk score, HCC patients were classified into low-risk and high-risk groups. The t-distributed stochastic neighbour embedding (t-SNE) was used to analyze the distribution of different subtypes using R packages "Rtsne". The Kaplan–Meier analysis (R package “survival”) was used for the prognostic role of risk signature. Besides, the “survminer,” “rms,” and “timeROC” R packages were applied to finish the receiver operating characteristic (ROC) analysis [48].

The ICGC and GEO datasets were used as validation datasets (test 1 and test 2). The risk score of HCC patients from ICGC and GEO datasets was calculated in the rain group, and the high/low-risk group was divided based on the median value of the risk score in the training group.

### 4.4 Independent prognostic analysis

By using univariate/multivariate Cox regression, we investigated the independent prognostic factors in the TCGA and ICGC datasets. Next, the nomogram model was performed to forecast the prognosis of HCC patients. Then, ROC and calibration curves were used to estimate the favourable accuracy of the nomogram model.

### 4.5 Immune microenvironment analysis

Single sample gene set enrichment analysis (ssGSEA, R “GSEABase”, “reshape 2” and “GSVA” packages), ESTIMATE (using R “ESTIMATE” packages), and CIBERSORT (https://cibersortx.stanford.edu/) were used for immune infiltration and immune functions as the previously described [49]. The correlation between immune and prognostic signature was analyzed.

IMMPORT database (ImmPort Portal) was used for the immune-related genes and the relationship between PRGs subtype/signature.

### 4.6 scRNA-seq analysis

The scRNA dataset of HCC was downloaded from GSE202642. The cells were filtrated by Seurat with 200< nFeature_RNA <7500 and percent.mt <10. The data was normalized, scaled and then was clustered using PCA analysis. The cells were identified using known marker genes as previously described [50]. Tumor cells ("EPCAM", "ALDH1A1"), epithelial cells ("KRT19", "CAPS", "KRT14"), B cells ("MS4A1", "CD79A"), T cells("CD3D", "CD3E"), NK cells ("FGFBP2", "FCG3RA"), myeloid-derived suppressor cells (MDSCs) ("ITGAM", "CD33"), monocytes or macrophages ("CD68", "CD163", "CD14"), DCs ("ITGAX"), Fibroblast ("ACTA2", "COL1A2", "DCN"), endothelial cells ("VWF", "PECAM1", "CLDN5"), Neutrophils ("S100A8", "S100A9", "CLCX12", "IL1B").

Subsequently, the subtype of T cells and macrophages were further analyzed using the following known marker genes [20,21].

T cells: Naive/CM CD4^+^ T (CCR7, CD4, SELL), T reg (FOXP3, IL2RA, CTLA4), Naive/CM CD8^+^ T (CD8B, CD8A), mucosal-associated invariant T cells (MAIT) (SLC4A10, KLRB1), cNK (FCGR3A), Cycling T (MKI67), APOC1+T (APOC1), γδT (TRDC, TRGC2) [20].

Macrophages: lipid-associated TAMs (LA-TAMs) (APOE, APOC1, ACP5), DC1 (CLEC9A), DC2 (CD1C, CD1E), pDC (LILRA4, GZMB), interferon-primed TAMs (IFN-TAMs) (CXCL10,PDL1, ISG15,S100A8, S100A10), immune regulatory TAMs (Reg-TAMs) (ARG1, MRC1, CX3CR1), inflammatory cytokine-enriched TAMs (Inflam-TAMs) (IL1B, CXCL1), proangiogenic TAMs (Angio-TAMs) (VEGFA, SPP1, VCAN, THBS1, FCN1), proliferating TAMs (Prolif-TAMs) (MKI67) [21].

Moreover, the cell-cell communication was analyzed using CellChat (http://www.cellchat.org/).

### 4.7 Statistical analysis

Statistical analysis was performed by R (version 4.1.1). The Wilcoxon test was used for the difference between HCC tissues and normal tissues, as well as low- and high-risk groups. The expression correlation was performed using person analysis. The *P*-value for statistically significant is as follows: *, *p* < 0.05; **, *p* < 0.01; ***, *p* < 0.001.

## Funding

This work was supported by the 10.13039/501100001809National Natural Science Foundation of China (Grant no. 31670838, 31370817).

## Data availability statement

Data will be made available on request.

## CRediT authorship contribution statement

**Chenjia Peng:** Conceptualization, Data curation, Methodology, Writing – original draft, Writing – review & editing. **Ying Wang:** Data curation. **Hengbo Zhang:** Data curation, Funding acquisition, Methodology, Writing – review & editing. **Ping Chen:** Conceptualization, Data curation, Funding acquisition, Methodology, Writing – review & editing.

## Declaration of competing interest

The authors declare that they have no known competing financial interests or personal relationships that could have appeared to influence the work reported in this paper.
