# Supplementary material for: The platelet-related genes associated with the prognosis of HCC by regulating cycling T cell and prolif-TAMs
Source: Heliyon. 2024 Feb 27;10(5):e26798. doi: 10.1016/j.heliyon.2024.e26798 (PMC10938119; doi:10.1016/j.heliyon.2024.e26798)
Supplement: Multimedia component 1 [file mmc1.docx]

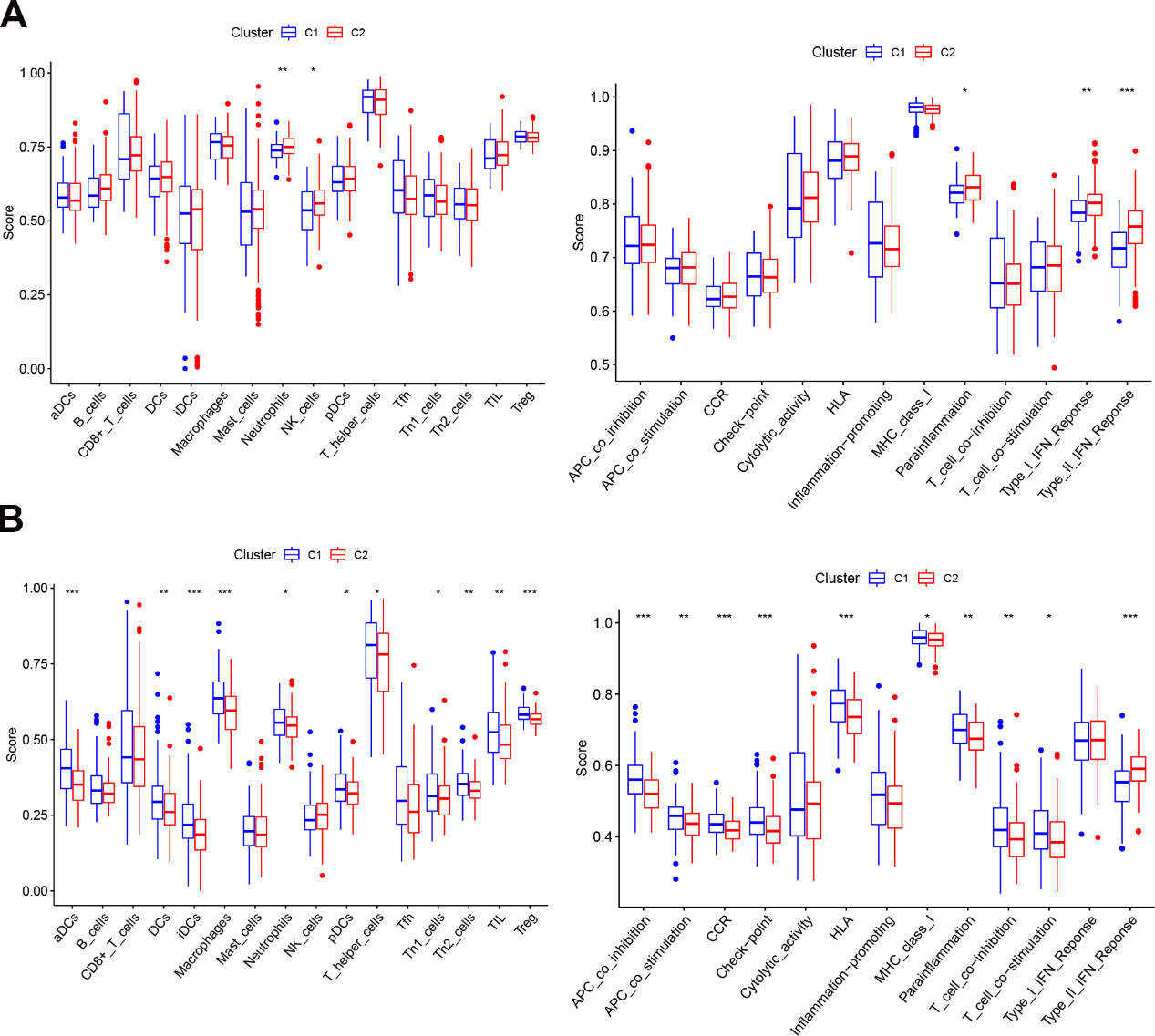


Figure S1. The immune analysis of HCC. A, The immune cell infiltration and immune function in HCC using TCGA dataset. B, The immune cell infiltration and immune function in HCC using ICGC dataset.


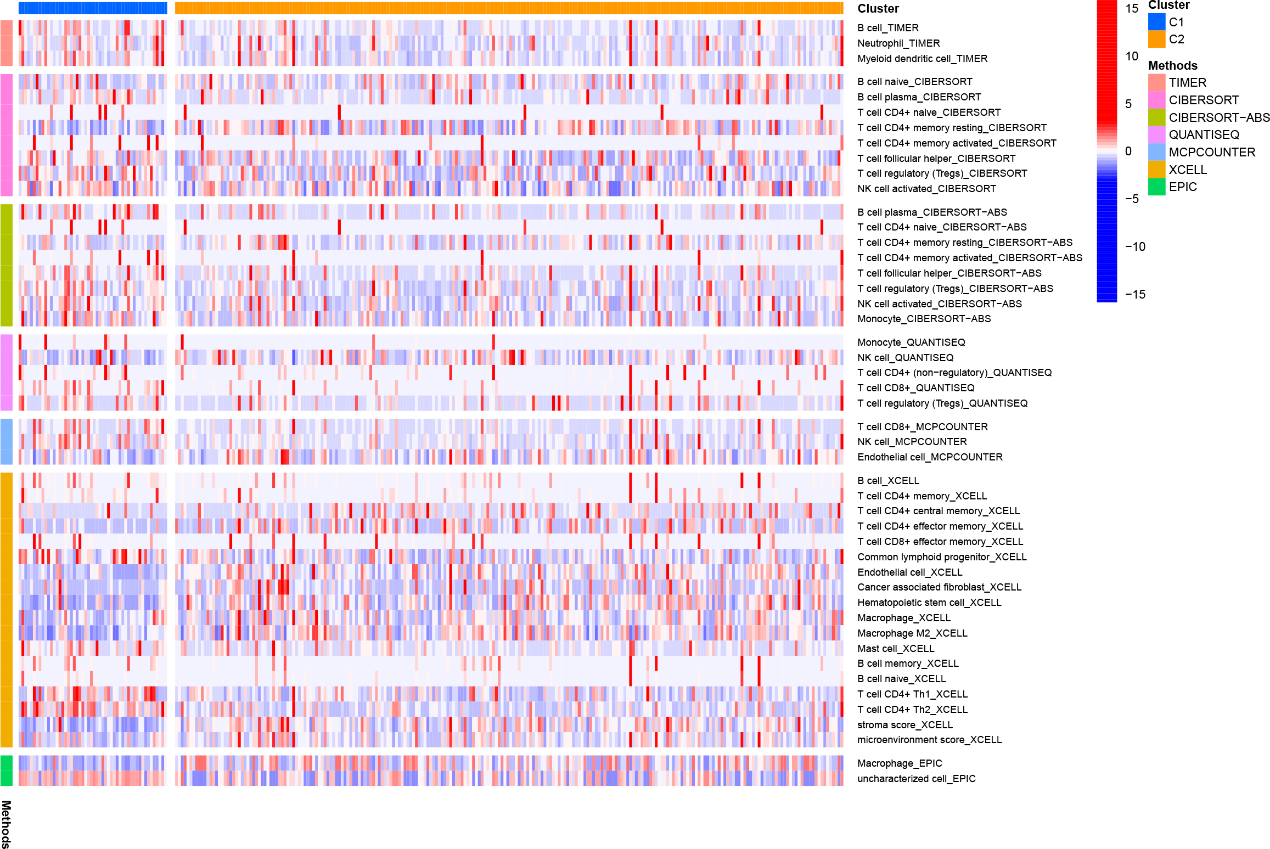


Figure S2. Heat map demonstrating immune cell infiltration in the C1 and C2 clusters in the TCGA databases.


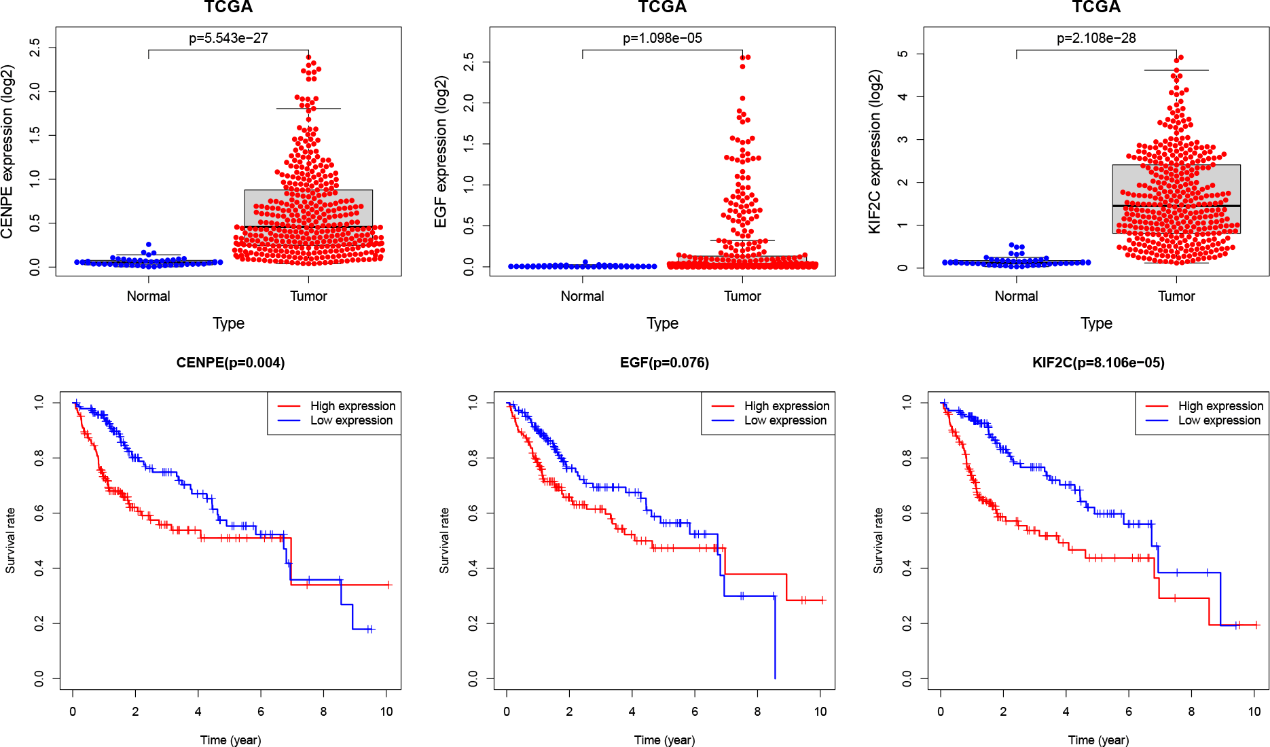


Figure S3. The expression and prognosis role of risk genes in the training TCGA dataset.


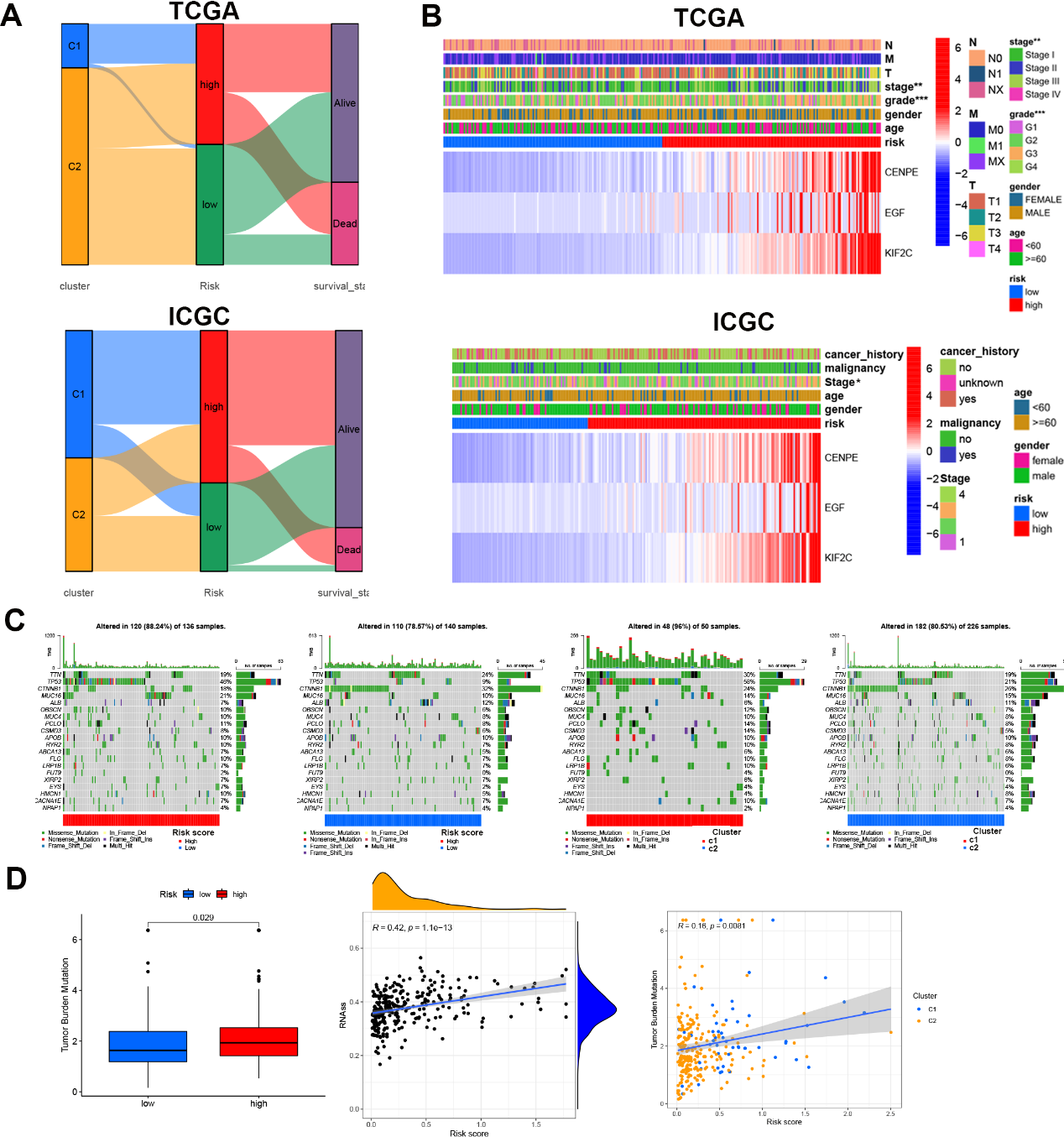


Figure S4. The relationship between PRGs subtype and PRGs risk signature. A, Alluvial diagram shows the correlation among subtypes, risk groups and survival status in TCGA and ICGC datasets. B, The heatmap of PRGs signature genes in TCGA and ICGC datasets. C, The waterfall plot revealed the mutation in different PRGs subtypes/signatures. D, The TBM and RNAss in different PRGs subtype/signature.


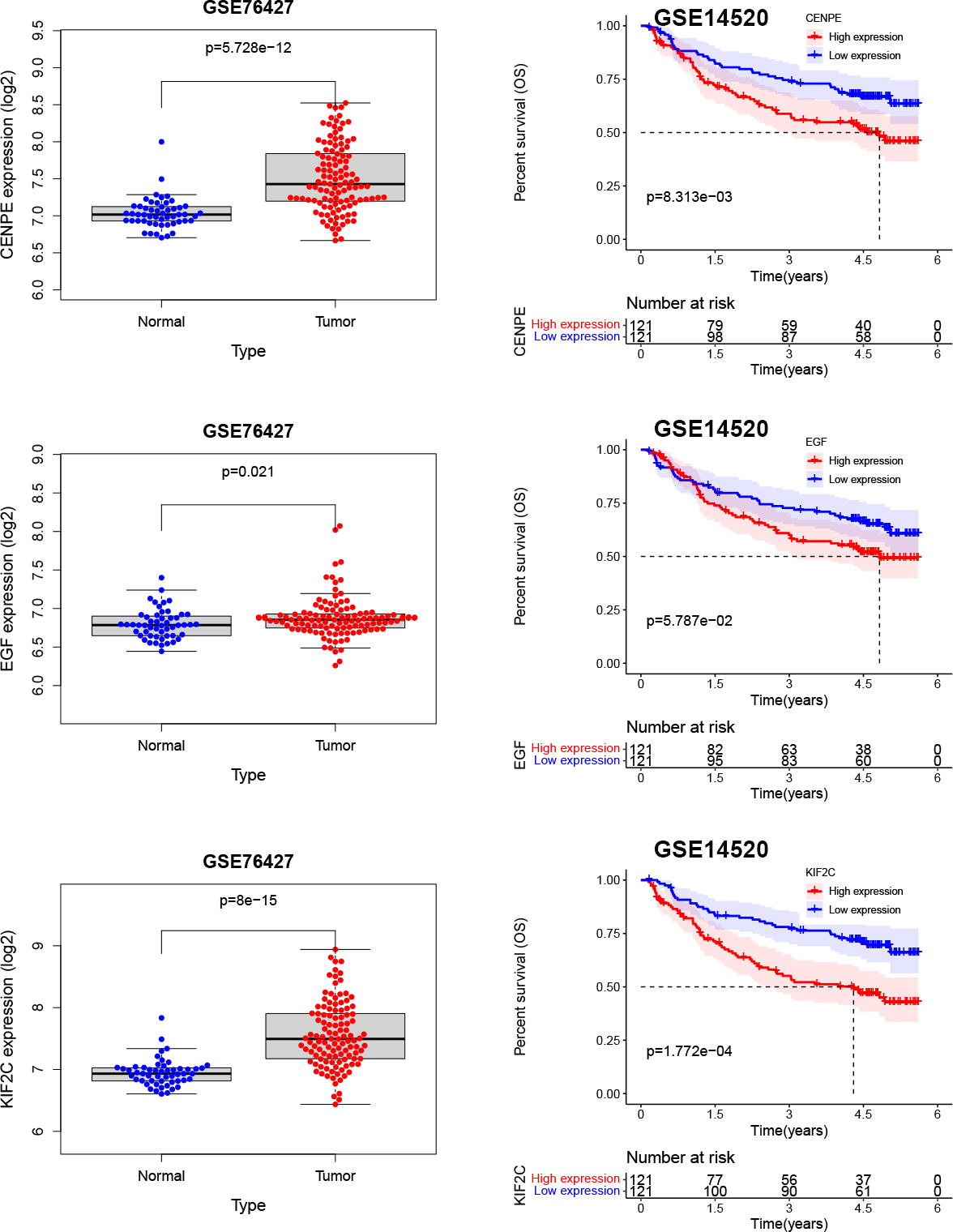


Figure S5. Expression and prognosis verification of PRGs signature genes using GEO datasets and qPCR analysis. A, Expression and prognosis verification of PRGs signature genes in GSE76427, GSE14520 datasets. B, The expression of PRGs signature genes in HCC cells. *, p<0.05; **, p<0.01.


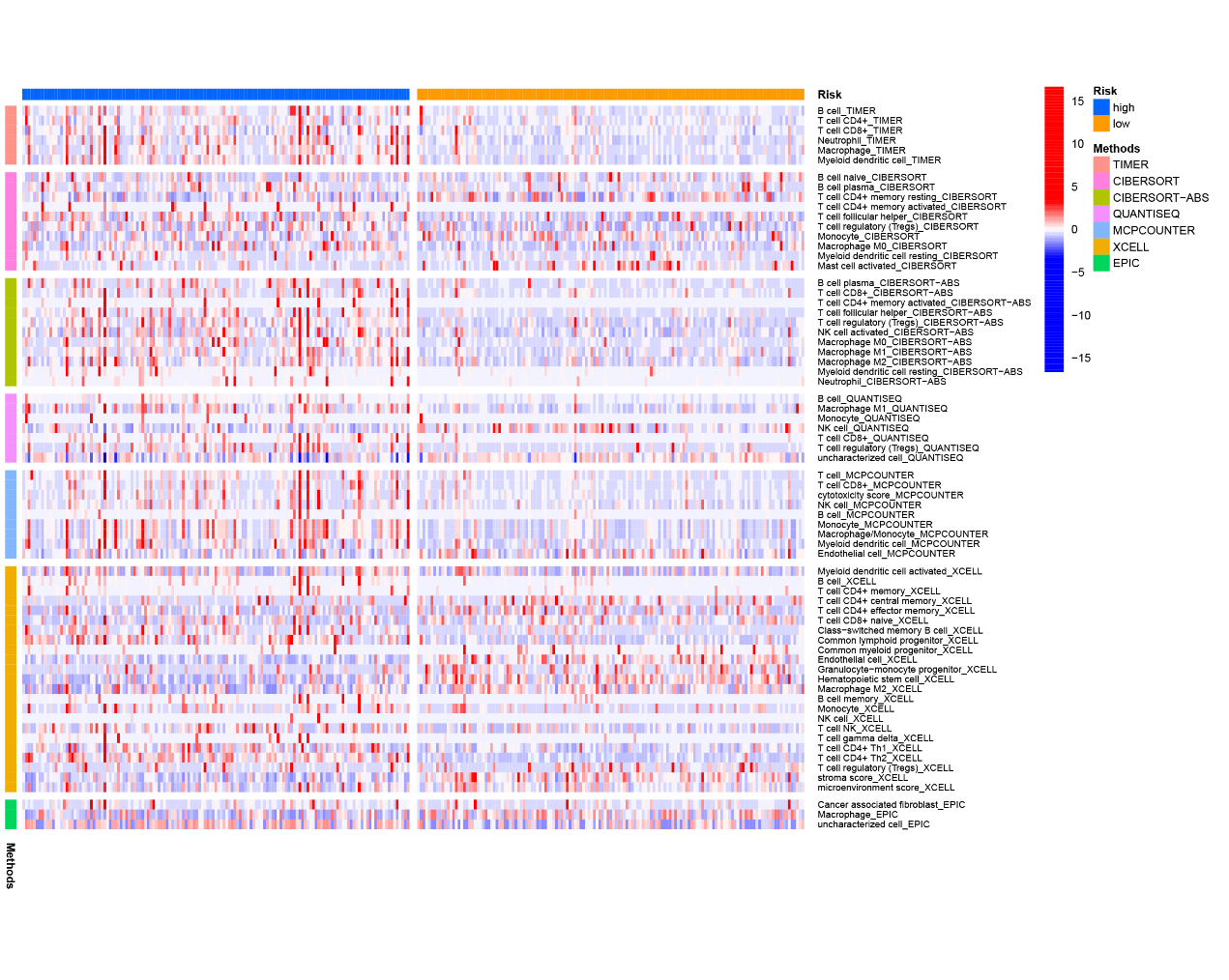


Figure S6.  Heat map demonstrating immune cell infiltration in the high- and low-risk groups in the TCGA databases.


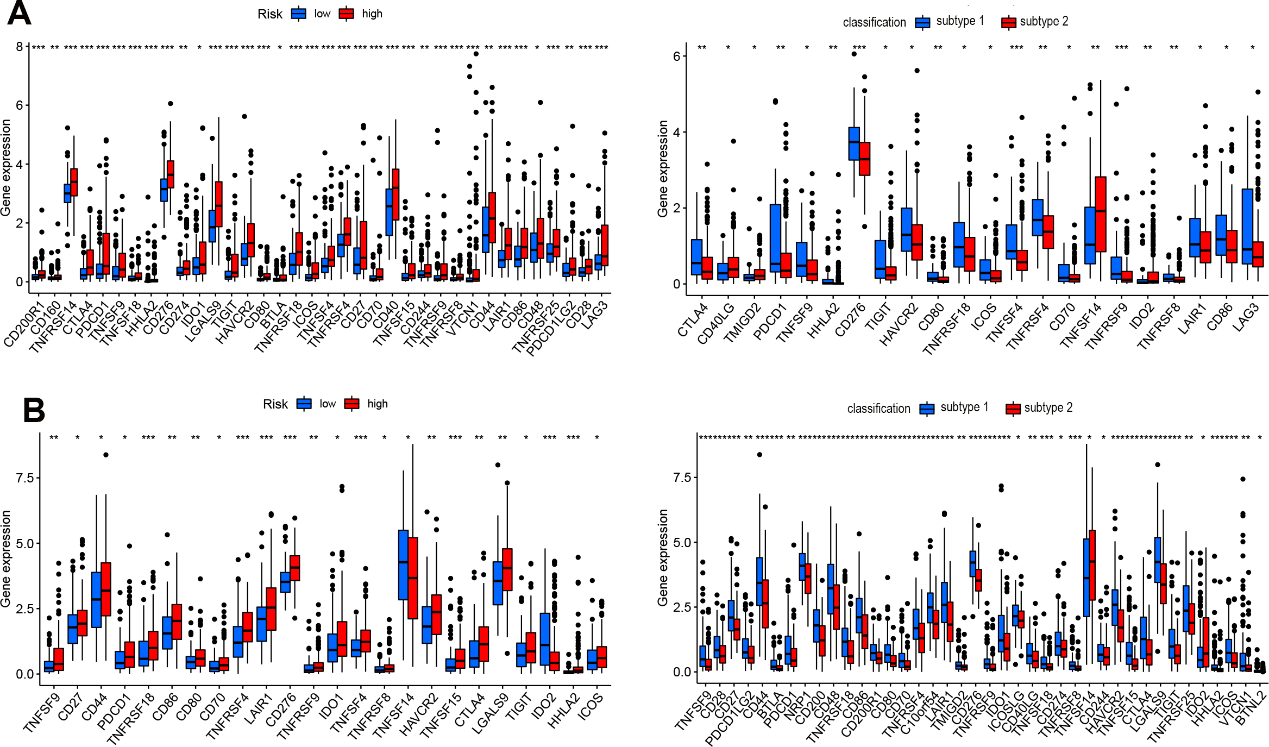


Figure S7. The immune checkpoint expression is associated with PRGs Subtypes and PRGs signature. A, The immune checkpoint expression in TCGA datasets. B, The immune checkpoint expression in IGCG datasets. *, p<0.05; **, p<0.01; ***, p<0.001.


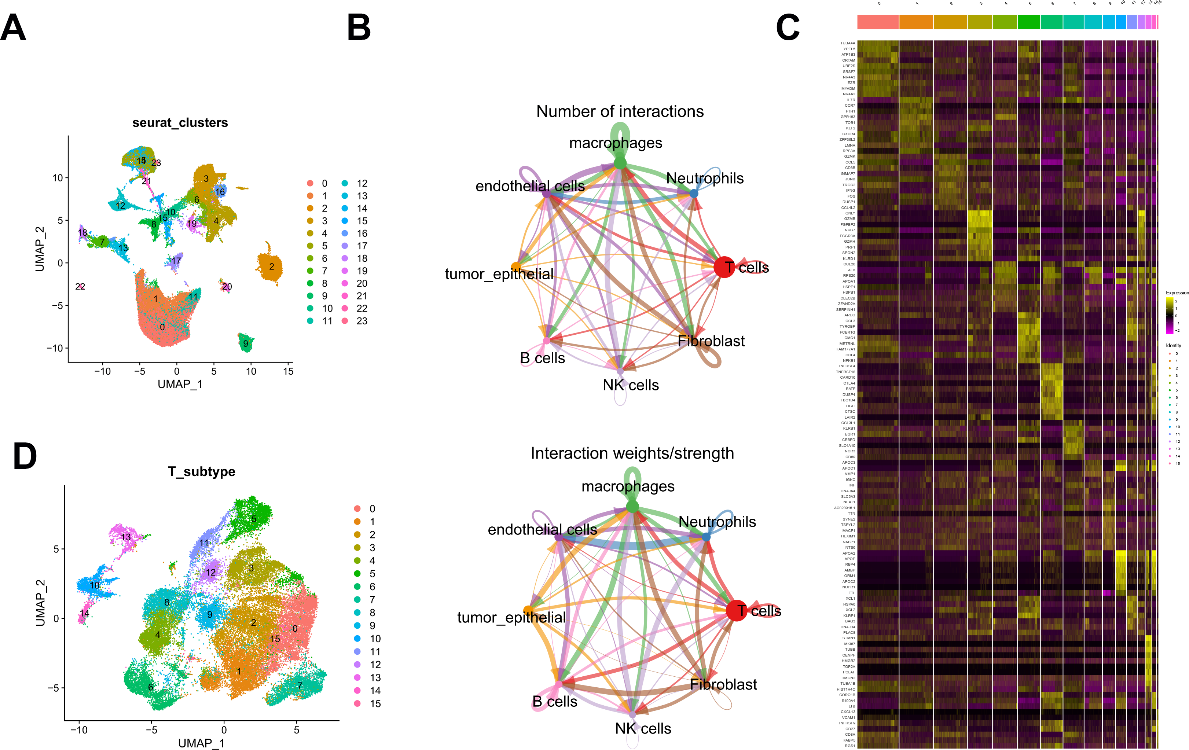


Figure S8. The scRNA transcriptomic analysis of HCC. The umap analysis of GSE202642. B, The cell-cell communication in HCC. C, The DEGs of cell clusters in HCC. D, The umap of T cells.


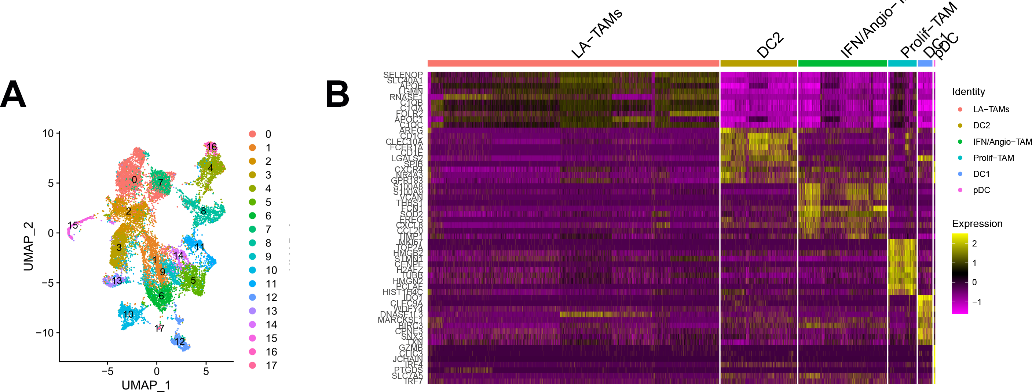


Figure S9. The subtype of macrophages. A, The umap of macrophage subtypes. B, The DEGs of macrophage subtypes.

Table S1. Platelet-related genes.

| A1BG | BCAR1 | CRK | DOCK9 | GATA6 | GP1BA | HDAC2 | ITPR2 | KIF3C | MAPK1 | PDLIM1 | PRKACG | RAF1 | SIN3A | TOR4A | VEGFA |
| --- | --- | --- | --- | --- | --- | --- | --- | --- | --- | --- | --- | --- | --- | --- | --- |
| A2M | BEX3 | CSK | ECM1 | GLA | GP1BB | HGF | ITPR3 | KIF4A | MAPK14 | PDPK1 | PRKAR1A | RAP1A | SNCA | TP53 | VEGFB |
| AAMP | BRPF3 | CTSA | EGF | GNA11 | GP5 | HMG20B | JAK2 | KIF4B | MAPK3 | PDPN | PRKAR1B | RAP1B | SNN | TPM1 | VEGFC |
| ABCC4 | CABLES1 | CTSW | EHD1 | GNA12 | GP6 | HRG | JMJD1C | KIF5A | MAX | PECAM1 | PRKAR2A | RAPGEF3 | SOD1 | TRPC3 | VEGFD |
| ABHD12 | CABLES2 | CTTN | EHD2 | GNA13 | GP9 | HSPA5 | KDM1A | KIF5B | MFAP3L | PF4 | PRKAR2B | RAPGEF4 | SOS1 | TRPC6 | VPS45 |
| ABHD6 | CALM1 | CXCL5 | EHD3 | GNA14 | GRB2 | IFNA1 | KIF11 | KIF6 | MFN1 | PF4V1 | PRKCA | RARRES2 | SPARC | TRPC7 | VTI1B |
| ABL1 | CALU | CYB5R1 | EIF2AK1 | GNA15 | GTPBP2 | IFNA10 | KIF12 | KIF9 | MFN2 | PFN1 | PRKCB | RASGRP1 | SPP2 | TSC22D1 | VWF |
| ACTB | CAP1 | CYRIB | ENDOD1 | GNAI1 | H2AC6 | IFNA13 | KIF13B | KIFAP3 | MGLL | PGRMC1 | PRKCD | RASGRP2 | SRC | TTN | WDR1 |
| ACTN1 | CAPZA1 | DAGLA | F13A1 | GNAI2 | H2BC21 | IFNA14 | KIF15 | KIFC1 | MICAL1 | PHACTR2 | PRKCE | RBSN | SRGN | TUBA1A | WEE1 |
| ACTN2 | CAPZA2 | DAGLB | F2 | GNAI3 | H3-3A | IFNA16 | KIF16B | KIFC2 | MLH3 | PHF21A | PRKCG | RCOR1 | STX4 | TUBA1B | WIPF1 |
| ACTN4 | CAPZB | DAPP1 | F2R | GNAQ | H3-3B | IFNA17 | KIF18A | KLC1 | MMD | PIK3CA | PRKCH | RGS10 | STXBP2 | TUBA1C | YPEL5 |
| ADRA2A | CARMIL1 | DGKA | F2RL2 | GNAS | H3C1 | IFNA2 | KIF18B | KLC2 | MMRN1 | PIK3CB | PRKCQ | RHOA | STXBP3 | TUBA3C | YWHAZ |
| ADRA2B | CBX5 | DGKB | F2RL3 | GNAT3 | H3C10 | IFNA21 | KIF19 | KLC3 | MPIG6B | PIK3CG | PRKCZ | RHOB | SYK | TUBA3D | ZFPM1 |
| ADRA2C | CCL5 | DGKD | F5 | GNB1 | H3C11 | IFNA4 | KIF1A | KLC4 | MPL | PIK3R1 | PROS1 | RHOG | SYTL4 | TUBA3E | ZFPM2 |
| AHSG | CD109 | DGKE | F8 | GNB2 | H3C12 | IFNA5 | KIF1B | KNG1 | MPP1 | PIK3R2 | PRUNE1 | RNF11 | TAGLN2 | TUBA4A | ZNF185 |
| AK3 | CD36 | DGKG | FAM3C | GNB3 | H3C13 | IFNA6 | KIF1C | LAMP2 | MYB | PIK3R3 | PSAP | RSU1 | TAX1BP3 | TUBA4B |  |
| AKAP1 | CD63 | DGKH | FCER1G | GNB4 | H3C14 | IFNA7 | KIF20A | LAT | MYLK | PIK3R5 | PTGS1 | RUFY1 | TBXA2R | TUBA8 |  |
| AKAP10 | CD9 | DGKI | FERMT3 | GNB5 | H3C15 | IFNA8 | KIF20B | LCK | NAP1L1 | PIK3R6 | PTK2 | RYBP | TEX264 | TUBAL3 |  |
| AKT1 | CDC37L1 | DGKK | FGA | GNG10 | H3C2 | IFNB1 | KIF21A | LCP2 | NFE2 | PIP4K2A | PTPN1 | SCCPDH | TF | TUBB1 |  |
| ALB | CDC42 | DGKQ | FGB | GNG11 | H3C3 | IGF1 | KIF21B | LEFTY2 | NHLRC2 | PLA2G4A | PTPN11 | SCG3 | TGFB1 | TUBB2A |  |
| ALDOA | CDK2 | DGKZ | FGG | GNG12 | H3C4 | IGF2 | KIF22 | LEPROT | NRGN | PLCG2 | PTPN12 | SELENOP | TGFB2 | TUBB2B |  |
| ANXA5 | CDK5 | DOCK1 | FHL1 | GNG13 | H3C6 | IRF1 | KIF23 | LGALS3BP | ODC1 | PLEK | PTPN6 | SELP | TGFB3 | TUBB3 |  |
| APBB1IP | CENPE | DOCK10 | FLNA | GNG2 | H3C7 | IRF2 | KIF25 | LHFPL2 | OLA1 | PLG | QSOX1 | SERPINA1 | THBS1 | TUBB4A |  |
| APLP2 | CFD | DOCK11 | FN1 | GNG3 | H3C8 | ISLR | KIF26A | LY6G6F | ORM1 | PPBP | RAB27B | SERPINA3 | THPO | TUBB4B |  |
| APOA1 | CFL1 | DOCK2 | FYN | GNG4 | HABP4 | ITGA2B | KIF26B | LYN | ORM2 | PPIA | RAB31 | SERPINA4 | TIMP1 | TUBB6 |  |
| APOH | CHID1 | DOCK3 | GAS6 | GNG5 | HBA1 | ITGB3 | KIF27 | MAFF | P2RY1 | PPM1A | RAB5A | SERPINE1 | TIMP3 | TUBB8 |  |
| APOOL | CLEC1B | DOCK4 | GATA1 | GNG7 | HBB | ITGB5 | KIF2A | MAFG | P2RY12 | PRDX6 | RABGAP1L | SERPINF2 | TLN1 | TUBB8B |  |
| APP | CLEC3B | DOCK5 | GATA2 | GNG8 | HBD | ITIH3 | KIF2B | MAFK | PCDH7 | PRKACA | RAC1 | SERPING1 | TMEM140 | VAV1 |  |
| ARRB1 | CLU | DOCK6 | GATA3 | GNGT1 | HBE1 | ITIH4 | KIF2C | MAGED2 | PCYOX1L | PRKACB | RAC2 | SH2B1 | TMSB4X | VAV2 |  |
| ARRB2 | COL1A1 | DOCK7 | GATA4 | GNGT2 | HBG1 | ITPK1 | KIF3A | MANF | PDGFA | RAD51B | RACGAP1 | SH2B2 | TMX3 | VAV3 |  |
| ASAH1 | COL1A2 | DOCK8 | GATA5 | HDAC1 | HBG2 | ITPR1 | KIF3B | MAP3K7CL | PDGFB | RAD51C | SHC1 | SH2B3 | TNFSF4 | VCL |  |

Table S2. The clinical information of HCC patients from TCGA database.

| clinical characters | group | number |
| --- | --- | --- |
| age | <60 | 165 |
|  | >=60 | 210 |
| gender | MALE | 254 |
|  | FEMALE | 121 |
| grade | G1 | 55 |
|  | G2 | 180 |
|  | G3 | 123 |
|  | G4 | 12 |
|  | unknow | 5 |
| stage | Stage I | 175 |
|  | Stage II | 86 |
|  | Stage III | 85 |
|  | Stage IV | 5 |
|  | unknow | 24 |

Table S3. The clinical information of HCC patients from ICGC database.

| clinical characters | group | number |
| --- | --- | --- |
| age | <60 | 44 |
|  | >=60 | 187 |
| gender | MALE | 170 |
|  | FEMALE | 61 |
| stage | Stage I/II | 141 |
|  | Stage III/IV | 90 |
